# In Vivo Quantitative Estimation of DNA-Dependent Interaction of Sox2 and Oct4 Using BirA-Catalyzed Site-Specific Biotinylation

**DOI:** 10.3390/biom10010142

**Published:** 2020-01-16

**Authors:** Arman Kulyyassov, Vasily Ogryzko

**Affiliations:** 1 Republican State Enterprise “National Center for Biotechnology” under the Science Committee of Ministry of Education and Science of Republic of Kazakhstan, 13/5, Kurgalzhynskoye road, Nur-Sultan 010000, Kazakhstan; 2 Institut de Cancerologie Gustave Roussy, UMR8126, Universite Paris-Sud 11, CNRS, 94805 Villejuif, France; vogryzko@gmail.com

**Keywords:** protein–protein interactions (PPI), proximity-utilizing biotinylation (PUB), biotin acceptor peptide (BAP), BirA, proteomics, liquid chromatography–tandem mass spectrometry (LC–MS/MS), multiple reaction monitoring (MRM), in vivo DNA-dependent protein–protein interaction, pluripotency transcription factors Sox2 and Oct4

## Abstract

Protein–protein interactions of core pluripotency transcription factors play an important role during cell reprogramming. Cell identity is controlled by a trio of transcription factors: Sox2, Oct4, and Nanog. Thus, methods that help to quantify protein–protein interactions may be useful for understanding the mechanisms of pluripotency at the molecular level. Here, a detailed protocol for the detection and quantitative analysis of in vivo protein–protein proximity of Sox2 and Oct4 using the proximity-utilizing biotinylation (PUB) method is described. The method is based on the coexpression of two proteins of interest fused to a biotin acceptor peptide (BAP)in one case and a biotin ligase enzyme (BirA) in the other. The proximity between the two proteins leads to more efficient biotinylation of the BAP, which can be either detected by Western blotting or quantified using proteomics approaches, such as a multiple reaction monitoring (MRM) analysis. Coexpression of the fusion proteins BAP-X and BirA-Y revealed strong biotinylation of the target proteins when X and Y were, alternatively, the pluripotency transcription factors Sox2 and Oct4, compared with the negative control where X or Y was green fluorescent protein (GFP), which strongly suggests that Sox2 and Oct4 come in close proximity to each other and interact.

## 1. Introduction

Protein–protein interactions (PPIs) play a fundamental role in many physiological processes, such as the cell division cycle or cell signaling in health and disease. The affinity and specificity of PPI are crucial, and alterations in them can lead to cellular malfunctions [1,2,3,4,5,6,7,8]. However, physical interactions between proteins are impacted by general protein–protein proximity (PPP) which arises as a result of aspects of intracellular order or their interactions via intermediates such as DNA [9]. Processes during the reprogramming of a cell to a pluripotent state at the molecular level include protein–protein proximity induced by binding to adjacent sites on DNA. The core pluripotency transcription factors (TFs) SRY-box 2 (SOX2), octamer-binding transcription factor 4 (OCT4), and NANOG lie in the core of the transcriptional network that controls stem cell pluripotency [10] and are key to inducing pluripotency in somatic cells [11,12,13,14,15,16]. For example, SOX2 binds to a sequence related to C(T/A)TTGTC, whereas OCT4 recognizes an octamer site with the consensus sequence ATGC(A/T)AAT. Mainly, they bind cooperatively to a composite motif formed by the juxtaposition of their individual binding sites [17], known as a “canonical” motif. Thus, the direct interaction between these key pluripotency transcription factors is DNA-dependent [18,19] and involves DNA-binding domains, such as the POU-specific domain (POU_S_) and a homeodomain (POU_HD_) of OCT4 and the high-mobility group (HMG) domain of Sox2. These proteins often bind to closely localized genomic sites, and their balanced expression as well as their precise mode of interaction with DNA at different time points are crucial for the pluripotency gene regulatory network (PGRN). Our knowledge about the plasticity of the PGRN is not complete, especially during early embryonic development or in the case of induced cell cultures. Recent papers described that quantitative properties of Oct4 and Sox2 such as expression levels or DNA-binding properties are predictive of cell fate decisions at the four-cell stage [20,21]. Oct4 and Sox2 were also found to play antagonistic roles in the differentiation of embryonic stem (ES) cells towards the mesendodermal (ME) and neuroectodermal (NE) fates [22]. Nevertheless, how the expression levels of Oct4 of and Sox2 change over time in naive ES cells, and whether these fluctuations bias germ layer cell fate commitment remain unknown.

Thus, methods that help to quantify DNA-dependable protein–protein interactions may be useful for understanding the mechanisms of pluripotency at the molecular level. Comprehensive studies of protein localization to specific chromatin sites provide plenty of valuable information on the transcriptional control of cells and the relationships between transcription factors. The hypothesis is that many of the proteins that bind in proximity to each other are in direct contact with one another, but this remains to be established both generally and for particular loci. Understanding how transcription factor assemblies change as cells move from one pluripotent state to another will allow us to view how the dynamic alterations in cell phenotype that underlie developmental transitions are ruled, which will definitely reinforce our ability to manipulate these cells to our will.

## 2. Materials and Methods

### 2.1. Design and Preparation of Constructs for Transient Expression

Primers containing N- or C-terminal part of the *Sox2* and *POU5F1* genes and XhoI or NotI restriction sites for subcloning into the vectors pcDNA3.1(+)-biotin acceptor peptide (BAP) and pOzFHHN-BirA (restriction sites XhoI, SalI, and NotI underlined):

Primer 1 ***Sox2_FD***: CACACACACTCGAGGGCATGTACAACATGATGGAGACGGA
Primer 2 ***Sox2_RS***: TGTGTGTGGCGGCCGCTCACATGTGTGAGAGGGGCAGTGT
Primer 3 ***Oct4_FD***: CACACACAGTCGACGGCATGGCGGGACACCTGGCTTCGGA
Primer 4 ***Oct4_RS***: TGTGTGTGGCGGCCGCTCAGTTTGAATGCATGGGAGAGCC

Since the *POU5F1* gene contains two XhoI sites inside of its open reading frame (ORF), for the design of primer3, an XhoI site was replaced with a SalI site, which generated compatible sticky ends. In order to enhance the flexibility between BAP (or BirA) and the protein of interest, we also added the additional codon GGC corresponding to glycine in the sequences of the forward primers 1 and 3.

The pcDNA3.1(+)-BAP-HP1γ and pOz-BirA-HP1γ vectors [23] were used for the constructions of new expression vectors, containing the genes *Sox2* and *POU5F1* fused with BAP and BirA. The insert sequences were confirmed by sequencing. The vector plasmids pcDNA3-BAP-Sox2 and pOz-humBirA-GFP are available from Addgene (Addgene ID 133281 and 133283, respectively).

### 2.2. Cell Culture, Transient Transfection, and Biotin Labeling In Vivo

#### 2.2.1. Materials

Stock solutions for cell culture, protein expression, and biotin labelling are detailed below. All solutions were sterilized by autoclaving or sterile filtration. Dulbecco’s Modified Eagle’s Medium (DMEM) medium containing 10% (*v*/*v*) fetal bovine serum (FBS) and 1× penicillin–streptomycin was prepared by adding 50 mL of FBS, South America origin (PAN biotech, Aidenbach, Germany), #P30-3306) and 4.5 mL of penicillin–streptomycin (100×) to 400 mL of DMEM medium. This medium can be stored at 4 °C for at least 1 month. For the preparation of the stock solution for biotin labeling (1 mg/mL), 0.5 mL of 410 mM NaOH was added to 50 mL of a suspension containing 50 mg of biotin (Sigma-Aldrich, MO, St. Louis, USA #B4501-1G) in water and vortexed until it was dissolved. A buffer solution of HBS 2× (200 mL) was used for the transfection, consisting of the following components: HEPES 2 g, KCl 0.15 g, glucose 0.4 g, NaCl 3.2 g, Na_2_HPO_4_ 0.0426 g (initial pH was 5.9, adjusted to pH 7.0 by addition in small portions of 1M NaOH solution, then filtered through a sterile filter from Millipore and stored at 4 °C). A Carl Zeiss Axioobserver A1 inverted research microscope was used for monitoring GFP expression and control the purity of the nuclear fractions during cell disruption.

#### 2.2.2. Methods

HEK293T cells were grown in DMEM (Gibco/Invitrogen, Thermo Fisher Scientific, Waltham, MA, USA) supplemented with 10% FBS South America origin (PAN biotech, Aidenbach, Germany) and 1× antibiotic (100 U/mL penicillin + 100 μg/mL streptomycin). One day before transfection, the monolayer of cells was trypsinized, and the cells were seeded in 10 cm culture dishes (approximately 1.6 × 10^6^ of cells per dish) in 5 mL complete DMEM supplement with 10% FBS at a density so that they reached approximately 80% confluence at the time of transfection. DMEM medium was changed at least 1 h before transfection. Eppendorf tubes (2 mL) marked as 0, 1, 2, 3, 4 were prepared, and plasmids were added in the following combinations and quantities:

pcDNA3-BAP-GFP, 3.0 µg; pcDNA3-BAP-Sox2, 5.0 µg; pOz-BirA-Oct4, 2.0 µg.
**0**—Control (No plasmid)
**1**—pcDNA3-BAP-GFP + pOz-BirA-Oct4 (for experiment with biotin pulse of 9 h)
**2**—pcDNA3-BAP-Sox2 + pOz-BirA-Oct4 (for experiment with biotin pulse of 9 h)
**3**—pcDNA3-BAP-GFP + pOz-BirA-Oct4 (for experiment with biotin pulse of 3 h)
**4**—pcDNA3-BAP-Sox2 + pOz-BirA-Oct4 (for experiment with biotin pulse of 3 h)

Then, 1.3 mL of sterile deionized water and 186 µL of 2M calcium chloride were added to each tube containing the plasmids. The plasmid solutions were added slowly, dropwise, to corresponding 15 mL Falcon tubes marked0, 1, 2, 3, 4, containing 1.5 mL of 2× HBS buffer, and mixed. The mixtures were incubated for 15 min at room temperature. The calcium phosphate–DNA precipitates were added to the HEK293T cells, mixing carefully by tilting the dishes, and the cells were incubated in a CO_2_ incubator for 48 h. For biotin labeling in vivo and, a stock solution of biotin (1 mg/mL) was added to a final concentration of 5 μg/mL for the specified time of labeling (in this experiment, 9 h and 3 h before harvesting the cells), while the pH was stabilized by the addition of 50 mM HEPES (pH 7.35) to the medium.

### 2.3. Cell Lysis and Sample Preparation

#### 2.3.1. Materials

Cytoskeleton (CSK) buffer was used for cell lysis and sample preparation and consisted of 100 mM NaCl, 300 mM sucrose, 10 mM Tris pH 7.5, 3 mM MgCl_2_, and 1mM EGTA (Santa Cruz Biotechnology, Heidelberg, Germany, #Sc-3593A). The buffer for cell disruption and nuclei isolation was prepared from the CSK buffer and contained 0.5% Triton X-100 (Sigma-Aldrich, St. Louis, MO, USA, #T9284), 1.2 mM phenylmethylsulfonyl fluoride (PMSF) (Sigma-Aldrich, #000000010837091001), and 1×protease inhibitor cocktail (Sigma-Aldrich, St. Louis, MO, USA, #P8340-5ML).

#### 2.3.2. Methods

In order to harvest HEK293T cells, DMEM medium was removed by aspiration, then 500 μL of PBS was added to each well, and the cells were resuspended, transferred to 1.5 mL Eppendorf tubes, and spun 5 min at 700 rpm. The supernatant was discarded. All subsequent steps were performed on ice; the cells were spun in a refrigerated centrifuge in the presence of PMSF and protease inhibitors in CSK buffer. Cell nuclei were isolated by disrupting the cells through pipetting in 150 µL of CSK buffer with 0.5% Triton X-100, followed by centrifugation for 5 min at 4000 rpm (4 °C). The purity of the nuclear fractions was monitored by light microscopy. The supernatant was discarded or, alternatively, it was transferred to a separate tube (cytoplasmic fraction) and kept along with the pellet which contained the nuclei. The pelletscan be stored at −20 °C for a few months. Then, 150 µL of CSK buffer was added to each pellet, and the lysates were sonicated with a microtip sonicator at 20 W for two cycles of 10 s each. One-tenth of the lysate (15 µL) was taken for Western blotting analysis. The solution was no longer viscous and was easily pipetted with a P-200 pipette. Cold acetone was added (−20 °C) to each tube to a final concentration of 80% *v*/*v*, and proteins were precipitated for 2–3 h at −20 °C. The supernatants were discarded after centrifugation at 1600 rpm for 20 min at 4 °C.

### 2.4. Ni-Sepharose Protein Binding, Purification, Propionylation, and On-Bead Digestion

#### 2.4.1. Materials

The 2× PAT buffer consisted of 20% glycerol, 40 mM Tris-HCl (pH-8.0), and 0.2% Tween (Sigma-Aldrich #P1379-100ML). For the preparation of buffer A (incubation buffer), 2.2 g of guanidine-HCl powder (Sigma-Aldrich, #50950-250G) was dissolved in 2 mL of 2× PAT buffer, and 200 µL of 5 M NaCl was added. The final concentration of guanidin-HCl was 6 M, that of NaCl was 250 mM, and the total volume of buffer A was 4 mL. For the preparation of buffer B (wash buffer), 1.8 mL of water was mixed with 2 mL of 2× PAT buffer and 200 µL of 5 M NaCl containing 0.2 mM PMSF, and the protease inhibitor cocktail was added. The final concentration of NaCl was 250 mM, and the total volume of buffer B was 4 mL. The following materials were used for on-bead propionylation and trypsin digestion: ammonium bicarbonate (NH_4_HCO_3_; Sigma-Aldrich, #09830-500G), Ni-sepharose 6 Fast Flow (GE Healthcare, Uppsala, Sweden, #17-5318-02), propionic anhydride (Sigma-Aldrich, #240311-50G), trypsin protease (MS grade, Thermo Fisher, Rockford, IL, USA #90057), trifluoroacetic acid (TFA, Sigma-Aldrich, #T6508-5ML). For the preparation of a trypsin stock solution, trypsin was dissolved in 1 mM TFA to a concentration of 100 ng/µL. This solution can be stored at −20 °C for a few months. The Eppendorf vacuum concentrator plus (#5305000304) was used to remove solvents and water from the samples. Keratin-free Eppendorf tubes and barrier tips were used to avoid the contamination of the samples with keratin. During sample preparation, nitrile gloves were worn before adding trypsin.

#### 2.4.2. Methods

Before adding buffer A, 1/10 of the samples was taken as INPUT. The nuclei pellet were resuspended in 0.5 mL of buffer A, rotated for 30 min at 4 °C, and then spun 1 min at maximum speed. The supernatants which contained the denatured proteins of interest were transferred to 1.5 tubes. Then, 150 µL of resin suspension was poured into the 1.5 tubes, which were spun for 5 min at 3000 rpm. The liquid was removed, and the beads in each tube were resuspended in 150–200 µL buffer A and spun again. The supernatants were discarded. Ni agarose beads were resuspended in 150–200 µL of buffer A, poured into each extract, rotated 2–3 h at 4 °C, and spun at 3000 rpm. The supernatants were kept as FLOWTHROUGH. The beads were washed 2 times with 150–200 µL of buffer A for 10 min at 37 °C and spun at 3000rpm. The supernatants were discarded. After this step, the resin was washed 2 times with 150–200 µL of buffer B for 10 min at 37 °C and spun at 3000 rpm. The supernatants were discarded. Then, the agarose beads were treated with 100 µL of 30% propionic anhydride in methanol and 40 µL of 50 mM NH_4_HCO_3_ for 1 h at 37 °C. In order to remove the excess of reagent, the resin was washed 2 times with 150–200 µL of 50 mM NH_4_HCO_3_for 10 min at 37 °C and 150–200 µL of acetonitrile and spun at 3000 rpm. The supernatants were discarded after each washing step. The beads were then dried and digested at 37 °C overnight using 0.4 µg of sequence-grade trypsin protease (MS grade, Thermo Fisher, Rockford, IL, USA) in 100 µL of 50 mM NH_4_HCO_3_. The next day, the tubes were spun at 3000 rpm, and the supernatants containing the tryptic digests were transferred to new Eppendorf tubes and placed in a vacuum concentrator to remove the solvent.

### 2.5. Desalting of Tryptic Peptide Mixturesby Ziptip

#### 2.5.1. Materials

Millipore Ziptips, micro-C18 (Sigma-Aldrich, #Z720003-96EA), were used for desalting the peptide mixtures. Other Ziptip kits such as C18 (Sigma-Aldrich, #Z720046-96EA) or C18 (ThermoFisher Scientific, Rockford, IL, USA #87782) can also be used in this protocol.

#### 2.5.2. Methods

The samples were adjusted to 10 µL of 0.1% of TFA, at a final pH < 4. In order to wet the sorbent medium, 5 µL of acetonitrile was aspirated into a Ziptip by moving the pipettor plunger up and down 7–8 times. The solvent was discarded. For equilibration of the sorbent medium, 5 µL of 0.1% TFA was aspirated into the Ziptip by moving the pipettor plunger up and down 7–8 times. The solvent was discarded. For maximum binding of the peptides’ mixtures to thee Ziptip pipette tip, the samples were aspirated and dispensed 7–8 times. Then, 5 µL of 0.1% TFA was aspirated into the Ziptip, and the sorbent medium was washed by moving the pipettor plunger up and down 7–8 times. The solvent was discarded. At this point, 5 µL of 0.1% TFA/50% acetonitrile elution solution was aspirated into the Ziptip and dispensed into a clean Eppendorf tube. The solvent was removed in a vacuum concentrator, resuspended in 15 μL of solution containing 0.1% TFA for mass spectrometry experiments, and transferred to an HPLC vial. The dried peptides can be stored at −20 °C.

### 2.6. LC–MS/MS Analysis

#### 2.6.1. Materials

Acetonitrile (ACN), LC/MS-grade (Carlo Erba, Val-de-Reuil Cedex, France #412342), and formic acid (FA) for mass spectrometry (Fluka, Steinheim, Germany, #94318-50ML-F) were used for the preparation of buffer A and buffer B as eluents during liquid chromatography. Buffer A: 0.1% FA; buffer B 90% ACN/10% in water, in 0.1% FA. The LC–MS/MS analysis was conducted with a nanoflow HPLC system (Thermo Dionex Ultimate 3000, ThermoScientific, Bremen, Germany) with an Acclaim PepMap100 C18 pre-column, 5 mm × 300 µm, 5 µm particles (Thermo Scientific, #160454), and an Acclaim Pep-Map RSLC column, 15 cm × 75 µm, 2 µm particles (Thermo Scientific, #164534). The MS/MS analysis utilized a QTOF Impact II mass spectrometer (Bruker, Bremen, Germany). Raw LC–MS/MS data were interpreted with the Bruker Compass Data Analysis (version 4.3) software (Bruker Daltonik GmbH, Germany).

#### 2.6.2. Methods

Degassed solvents were used for nanoLC (mobile phase (A): 0.1% (*v*/*v*) FA in water; organic phase (B) 0.1% (*v*/*v*) FA in ACN). The vials with the samples (15 µL) were placed into a rack and put onto the Dionex Ultimate 3000 RS Autosampler.

### 2.7. Creating the Multiple Reaction Monitoring Method

For creating the multiple reaction monitoring (MRM) method, a default application method was used as a template (targeted protein quantification middle-band CID-MRM.m).

- In the otofControl 4.0 program (Bruker Daltonik GmbH, Germany), the method named “Targeted protein quantification middle-band CID-MRM.m” was selected, and then for global settings, the spectra rate was set to1.0 Hz;
- The mass range of the MS scan was set to extend from *m*/*z* 200 to 1300 in positive ion polarity mode;
- In the Source page of the system configuration pane, nanoBooster box was selected, and 1300 V for the capillary, 3.0 L/min for dry gas, and 150 °C for dry temperature were chosen;
- In the MRM subpage of the MS/MS page, the *m*/*z* values of propionylated BAP (563.2) with collision energy 27.0 eV and biotinylated BAP (648.8) with collision energy 33.0 eV were added. Mass width was set to3.00;

After the creation of the MRM method, the Hystar 3.2 program was loaded, and the sample table and the open template file were selected. In the General page, we specified a subdirectory for Result Data Path, added a Sample Identifier and injection volume, and selected a vial position in the tray for each line. In the Method page, LC Method Part and MS Acquisition Method Part for each line were specified and saved in the sample table. After adding all data, we clicked the Start acquisition button on the menu. A Data Analysis program was used to validate the presence of the targeted tryptic peptides by first ensuring the corresponding MRM transitions and MS/MS spectra. This program also allows the integration of the peak areas of the different MRM transitions, which was used to determine the ratios between the peak areas of the tryptic peptides in all samples for quantification.

## 3. Results and Discussions

### 3.1. Overview of the Technique

**Figure 1:**
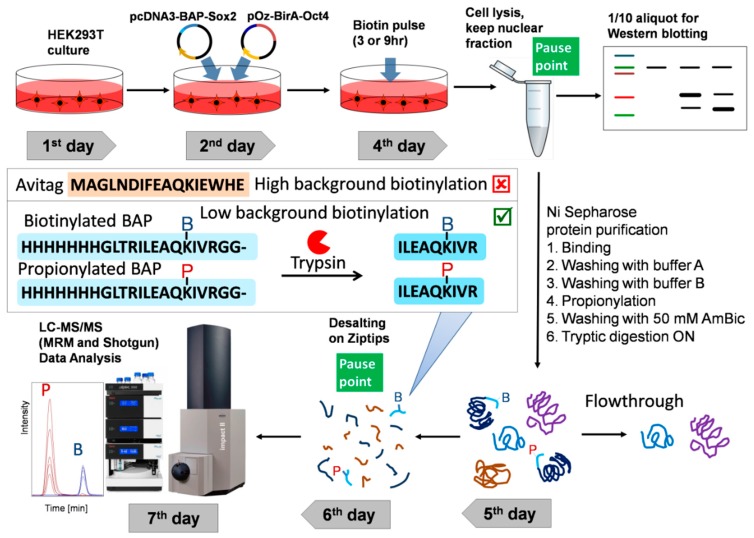
Experimental workflow for the detection and quantitative analysis of protein–protein interactions (PPI) between pluripotency transcription factors. Cells expressing biotin acceptor peptide (BAP)-Sox2 and BirA-Oct4 were labelled by biotin (3 h or 9 h) before harvest. The cells were lysed in cytoskeleton (CSK) buffer with 0.5% Triton X-100 to isolate the nuclei. The nuclear fractionsere incubated on Ni-sepharose beads to purify and enrich the fused proteins. The Ni-sepharose-bound proteins were treated by propionic anhydride, washed, and digested on the beads with trypsin, and the tryptic peptides were desalted by Ziptips. The peptide mixtures were then analyzed by LC–MS/MS. The propionylated and biotinylated BAP peptides were identified and quantified using Data Analysis software.

The optimized workflow for the quantitative analysis of in vivo protein–protein interactions (proximity) is depicted in Figure 1. HEK293T cells were transfected with the two plasmids pcDNA3-BAP-Sox2 and pOz-BirA-Oct4 using the calcium phosphate protocol. Before harvesting, the cells were labeled by adding biotin to the DMEM medium (3 h or 9 h biotin pulses). The cells were subsequently lysed and centrifuged, and the nuclear fraction was sonicated. One-tenth of an aliquot of each sample was used for Western blotting analysis of 7× His-tagged and biotinylated proteins. The recombinant proteins were enriched in chaotropic buffer with Ni-sepharose resin by means of the His-tag on the BAP-Sox2 construct. In order to label nonbiotinylated proteins BAP-Sox2, the beads were treated with propionic anhydride. Propionylation was used to protect the nonbiotinylated BAP peptide from tryptic cleavage on the target lysine. Such an approach allows one to obtain modified and nonmodified peptides of comparable sizes, facilitating the interpretation of the results. On-bead protein digestion is preferable over in-gel protein digestion, because the on-bead workflow significantly reduces the number of fractions to be measured by mass spectrometry, as compared with in-gel digestion. After desalting on Ziptip, the peptide mixtures, containing biotinylated and nonbiotinylated BAP peptides, were analyzed by LC–MS/MS, using the Bruker Compass Data Analysis software (Bruker Daltonik GmbH, Germany).

### 3.2. Experimental Design

Two proteins can be expressed from a single bicistronic vector that expresses one mRNA encoding both BirA and the target BAP-fused proteins. However, such bicistronic design may increase the local concentration of the enzyme in the vicinity of the target, leading to more efficient biotinylation [24], and this design cannot be applied for the estimation of protein–protein interactions in physiologically relevant context. Thus, in vivo biotinylation based on the co-expression from different plasmids of a protein of interest fused to BAP together with the biotin ligase BirA fused to an interaction partner was chosen as an alternative system. To achieve a more efficient biotinylation of the target protein, human-optimized BirA was used [25]. As a negative control, a BAP-tagged irrelevant protein, known to have no interaction with the pluripotency transcription factors, wasused. To this purpose, GFP is a good choice, as it can also serve to control the transfection efficiency. To minimize nonspecific binding to the affinity resin, it is recommended to perform binding and washing of the beads at least twice with a chaotropic buffer. We recommend using 6 M guanidine hydrochloride instead of 8M urea, since urea can lead to carb amylation of peptides and proteins and, subsequently, incomplete digestion of proteins [26]. On-bead protein digestion is preferable to in-gel protein digestion, because the on-bead workflow significantly reduces the number of fractions to be measured by mass spectrometry, as compared with in-gel digestion, while still identifying the most distinct interactors. Propionylation was used to protect the nonbiotinylated BAP peptide from tryptic cleavage of the target lysine. This method has been widely used elsewhere, for example, in the analysis of histone modifications [27,28]. Such an approach allows one to obtain modified and nonmodified peptides of comparable sizes, facilitating the interpretation of the results. The digested peptides were analyzed using a nanoreverse-phase HPLC system coupled to a QTOF mass spectrometer. The instrument was used in two modes: targeted MRM analysis for the relative quantification of biotinylated and propionylated BAP peptides and shotgun (or discovery) mode for the identification of all proteins nonspecifically bound to the resin (Supplementary Tables S1–S4). The raw data obtained from MS were examined using the Data Analysis program by performing extracted ion chromatograms (EIC) of the precursor ions at 563.2001 *m*/*z* (propionylated BAP) and 648.4190 *m*/*z* (biotinylated BAP) and calculating the areas for fragment ions *y*_8_, *y*_7_, *y*_6_, *y*_5_, and *y*_4_. To predict the *m*/*z* values of the fragment ions, we used an accessible online program that allowed us to calculate these values for *y* and *b* ions for both propionylated and biotinylated BAP peptides (http://db.systemsbiology.net/proteomicsToolkit/FragIonServlet.html). The raw data were processed by the Data Analysis program by using the “protein analysis” function on the menu to generate an mgf file. The mgf file was used to perform a “target-decoy” search on the Mascot server, applying 1% false discovery rate (FDR) for peptide spectrum matches (PSMs) above the homology threshold (Supplementary Tables S1–S4). The MS proteomics data have been deposited into the ProteomeXchange Consortium via the PRIDE partner repository, with the data set identifier PXD015756.

### 3.3. Development of the Protocol

This chapter describes the application of the proximity-utilizing biotinylation (PUB) method [23,29] to detect the protein–protein proximity of the transcription factors Sox2 and Oct4 in vivo. We chose Oct4 and Sox2 proteins because they are the primary regulators of pluripotency and also to demonstrate that PUB can detect transient PPI, which cannot be accomplished by other methods. For example, the list of Oct4 interactors identified by co-immunoprecipitation (Co-IP) lacked one of the best-studied partners of Oct4, namely, Sox2 [30]. This is probably because Oct4 and Sox2 interact more stably when bound to adjacent sites on DNA. It is therefore pertinent to ask which transcription regulators colocalize when bound to DNA. We adapted this method for the study and quantitative estimation of in vivo PPI betweenBAP-Sox2 and BirA-Oct4.

**Figure 2:**
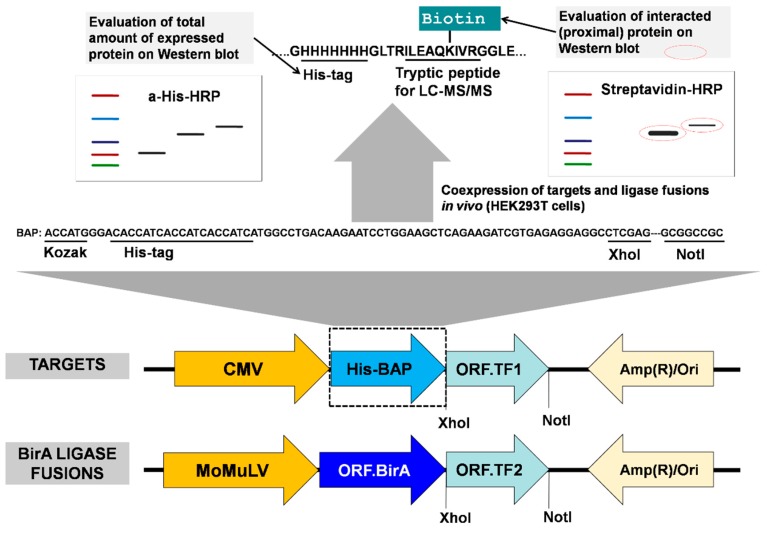
Design and features of the system. Two candidate interaction proteins, for example, two transcription factors TF1 and TF2, one of which is the target fusion protein BAP-TF1, and the other is the BirA ligase fusion protein BirA-TF2, are coexpressed in cells (for example HEK293T or HeLa). The cells are pulse-labeled with biotin. If there is interaction (top, right western blot), the BAP-TF1 fusion protein is more efficiently biotinylated (encircled red-dotted lined bands). The positions of the CMV/MoMuLVenhancers and of the BAP and BirA sequences, relative to the cloned ORF, are indicated. Nucleotide and amino acid sequences encoding the designed His-BAP are shown.

The principle of the method is the enzyme/substrate pair reaction [31,32,33], where two proteins to be tested for their proximity in vivo are coexpressed in cells (for example HEK293T or HeLa), one as fused to the enzyme BirA, and the other fused to aBAP. A more efficient biotinylation of the BAP is expected when the two proteins are in proximity to each other, for example, when an interaction occurs. The biotinylation status of the BAP fusion protein can be further monitored by Western blot or mass spectrometry. To implement this principle, we constructed two types of vectors, one for the expression of the BirA fusion protein, and the other for the expression of the BAP fusion protein, (Figure 2). All BirAfusion vectors contained the humanized version of the *Escherichia coli* biotin ligase [25]. Each vector was constructed in two forms, with protein expression regulated by either a strong cytomegalovirus (CMV) enhancer (plasmids based on pcDNA3.1(+)) or a weaker moloney murine leukemia virus (MoMuLV) (plasmids based on pOz) enhancer. In this work, the MoMuLV enhancer was used for the expression of BirA fusion proteins, while the BAP fusion proteins were expressed from vectors containing the stronger (CMV) promoter. This setting typically allowed us to achieve an excess of BAP fusion proteins (BAP-TF1 or biotinylation target) over BirA fusion proteins (BirA-TF2 or biotinylating enzyme), which was essential for further analysis.

The His-tag in BAP is used to allow enrichment on Ni-sepharose beads or help to evaluate and normalize the total amount of expressed recombinant proteins by Western blot. After adding biotin to the medium, when BAP-TF1 and BirA-TF2 bind proximal sites on DNA, the first protein is biotinylated by the second ligase fusion protein. Ahigh level of biotinylation of the target indicates the interaction (or proximity) of the two proteins, which can be quantitatively evaluated by densitometric analysis of Western blots or measured by LC–MS/MS. Also, as we demonstrated earlier [23], the newly designed BAP has a lower background biotinylation level in comparison with Avitag (Figure 1).

**Figure 3:**
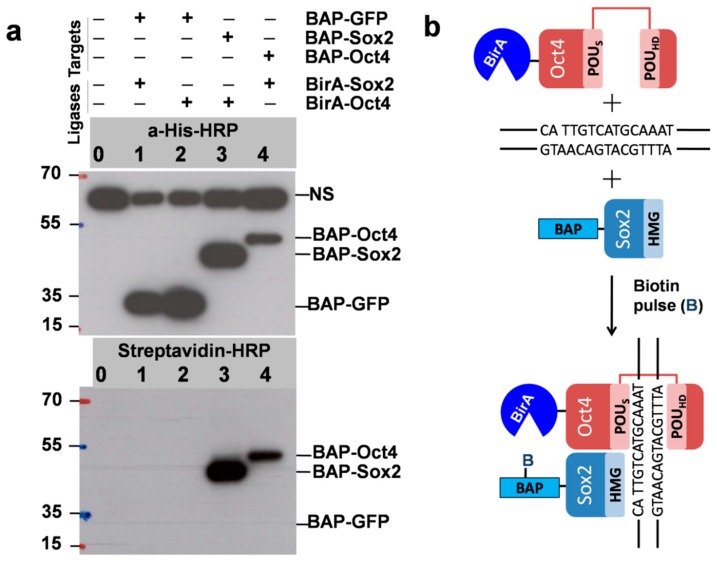
(**a**) Biotinylationis interaction/proximity-dependent (DNA-dependent binary interaction between Sox2 and Oct4). The positions of the BAPfusion proteins and nonspecific signal (NS) are indicated. Importantly, the NS was also detected in untransfected cells and corresponds to cellular proteins that bind to the anti-His antibodies. Expression of BAP-GFP was used as a control (since GFP does not have a DNA-binding domain). Western blot using anti-His-HRP shows the total expression levels of the recombinant proteins BAP-GFP, BAP-Sox2, and BAP-Oct4 (Top, lanes 1–4). Strong signals in the streptavidin-HRP Western blot identifyproteins which are in proximity in vivo (lanes 3, 4, Bottom). Biotin pulse, 30 min. (**b**) Since the high-mobility group (HMG) domain of BAP-Sox2 and the POU domain of BirA-Oct4 assemble on closely spaced composites of CATTGTC-like and ATGCAAAT-like DNA binding sites, the BAP target will be biotinylated. No biotinylation was observed when coexpressing BAP-GFP and BirA-Sox2 and BAP-GFP and BirA-Oct4 (lanes 1 and 2).

As mentioned, to test the PUB method, we chose Sox2 and Oct4 pluripotency transcription factors as the model proteins TF1 and TF2 and prepared vectors in which these genes were fused with BAP and BirA. The plasmids were used for transient transfection of HEK293T cells in six-well plates. As expected, biotinylation was detected by Western blot for cells expressing BAP-Sox2 and BirA-Oct4 (lane 3 in Figure 3a), which was surprisingly very strong. The same was observed for cells with the reverse combination BAP-Oct4 and BirA-Sox2 (lane 4).

Despite the presence of a comparable amount of BAP-GFP (used as a control), as indicated by Western blot with anti-His-HRP, no biotinylation was observed by Western blot with streptavidin-HRP (lanes 1 and 2). This was probably also due to the short biotin pulse time of 30 min. We used longer biotin labeling times (3 h and 9 h) in subsequent experiments to estimate the biotinylation level in the control which is the result of random collisions between BAP-GFP and BirA-Oct4. As it was mentioned in the introduction the HMG domain of BAP-Sox2 and the POU domain of BirA-Oct4 assemble on closely spaced DNA binding sites, which resulted in the biotinylation of the BAP target and a strong signal in the streptavidin Western blot (Figure 3b). The quantitation of the protein–protein interaction of these transcription factors was assessed by densitometric analysis of the Western blots.

This method allows also a study of the biotinylation levels of proteins that have different subcellular locations, e.g., the nucleus and the cytoplasm. A lysis buffer containing 0.5% Triton is known to disrupt cells, producing two fractions, and the purity of the nuclear fraction can be monitored by a light microscope [34]. Although Sox2 and Oct4 are mainly present in the nucleus [15], fusion proteins with BAP or BirA may alter their localization in the cell. In order to address this issue, we also analyzed the cytoplasmic fraction of HEK293T cells expressing BAP-Sox2 and BirA-Oct4. The Western blots of the cytoplasmic fractions showed no presence of BAP-Sox2 in the cytoplasm as demonstrated by anti-His-HRP blots (Supplementary material, Figure S1).

More unambiguous results could be obtained with the use of another nuclear protein as a control instead of GFP. As a model protein, we chose Tap54beta (or RuvB-like2) [35,36] and coexpressed BAP-Tap54beta and BirA-Oct4. Although this protein was present in comparable amounts in the nuclus and cytoplasm as shown by anti-His-HRP blots, weak biotinylation of the BAP-Tap54beta protein was observed in the nuclear fraction, and no noticeable biotinylation signal was found in the supernatant (Supplementary material, Figure S1). These results indicate that BirA-Oct4 is mainly localized in the nucleus and, in principle, it could also biotinylate BAP-Tap54beta as a result of random collision; however, the biotinylation level of BAP-Tap54beta was much lower in comparison to that obtained when coexpressing the pair BAP-Sox2 and BirA-Oct4. It should be noted that we also used different transient transfection protocols for the described experiments. Along with the classical calcium phosphate method whose buffers can be made in the lab from accessible and cheap reagents, commercial FuGENE or Lipofectamine were applied for the expression of target and BirA fusion proteins of interest.

Since mass spectrometry provides a more accurate quantification, a protocol based on a modified workflow reported earlier [23] was used, in which the SDS-PAGE step was eliminated, and His-tagged proteins from the cell lysates were purified in 6 M guanidine HCl buffer on Ni-sepharose beads. After several washing steps, propionylation, and on-beads digestion, each peptide mixture was run twice on LC–MS/MS. The QTOF instrument was set to the MRM mode in the first run and to the shotgun mode in the second run. The shotgun method was used to identify abundant proteins nonspecifically bound to the beads after washing. BAP-Sox2 and BAP-GFP fusion proteins were also identified, among other abundant proteins, and highlighted in yellow (Supplementary Tables S1–S4).

For samples derived from cells expressing BAP-GFP, we used the taxonomy filter “All entries”, and for samples derived from cells expressing BAP-Sox2, the taxonomy filter “*Homo sapiens* (human)” in Mascot search engine. More washing steps with guanidine hydrochloride (Gu∙HCl) decrease the level of nonspecific binding to Ni-sepharose; however, as we demonstrated, two washings with Gu∙HCl were sufficient to get clear EIC in the MRM experiments for the estimation of the biotinylation level of BAP.

### 3.4. Quantitative Evaluation of DNA-Dependant Interactions of Sox2 and Oct4

**Figure 4:**
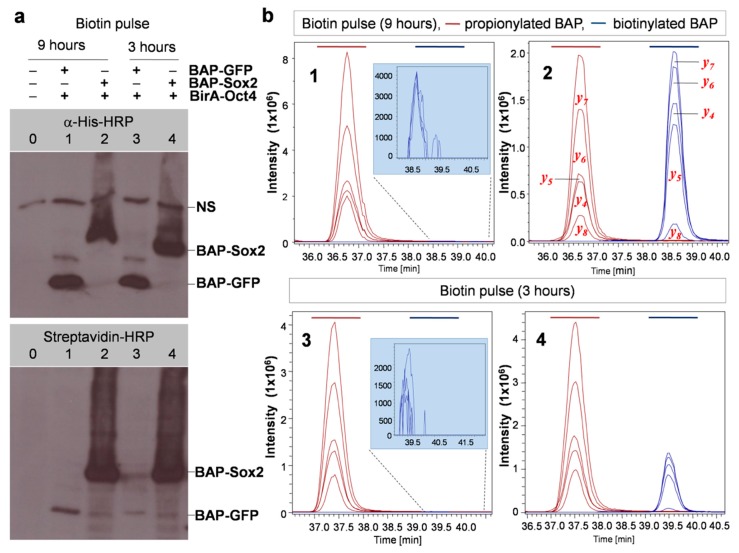
The biotinylation levels are interaction/proximity-dependent. (**a**) Western blotting to detect the interaction of pluripotency transcription factors versus a control at two biotin pulse times. Combinations of BirA and BAP fusion proteins were transfected separately into cells: lanes 1 and 3: BAP-GFP + BirA-Oct4 (control), lanes 2 and 4: BAP-Sox2 + BirA-Oct4, 0: nontransfected cells, NS: nonspecific signal. (**b**) Multiple reaction monitoring (MRM) detection of propionylated and biotinylated BAPs. Shown are extracted ions chromatograms for the most intensive fragmentation ions present in the MS/MS spectra of the respective peptides (y-series). Note the change of peaks corresponding to biotinylated BAP (colored blue) relative to propionylated BAP (colored brown) at different biotin pulse times (sample 2 and sample 4). Numbers in the graphs correspond to the lane numbers in the Western blots.

**Figure 5:**
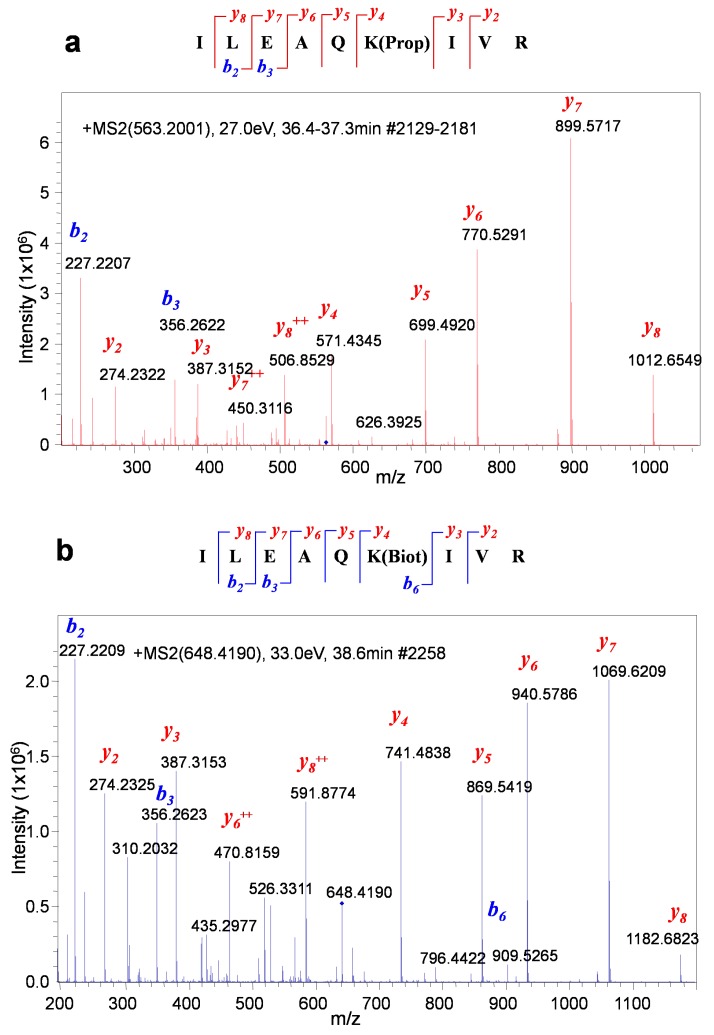
Propionylated and biotinylated BAP peptides (ILEAQK(propionyl)IVR and ILEAQK(biotinyl)IVR) can be unambiguously differentiated by MS/MS. (**a**) MS/MS spectrum of a propionylated BAP peptide. (**b**) MS/MS spectrum of a biotinylated BAP peptide.

The results that we obtained using this protocol with the model system BAP-Sox2 + BirA-Oct4 with different biotin pulse times are shown in Figure 4. From this figure, it is evident that the biotinylation level notably increased when the cells were labeled with biotin for longer times (9 h vs. 3 h). There was also a huge difference in the biotinylation levels in comparison with the control (BAP-Sox2 vs BAP-GFP), as shown by the Western blots (Figure 4a) and EIC. It is also noticeable that a biotinylation signal for of BAP-GFP was observed, as indicated by Western blotting results for samples 1 and 3 (bottom blot, Figure 4a) and EIC (drawing tab highlighted in blue in Figure 4b), which appeared to be due to random collisions between BAP-GFP and BirA-Oct4, while no biotinylation was detected when performing a short biotin pulse 30 min (Figure 3, lanes 1 and 2). Using the MRM method, it is possible to make a relative quantification of biotinylated versus nonbiotinylated BAP peptides (Figure 5). A comparison between total ion chromatograms (TIC) of the biotinylated and propionylated BAP in LC–MS/MS data cannot be done directly, as the ionization efficiency generally depends on the chemical structure of a molecule and would thus be different for propionyl and biotin residues. Thus, for recalculation of the biotinylated BAP/total BAP ratio, we used the ionization coefficient k=11.9, which was estimated earlier from SILAC experiments [23]. After recalculation, the data were normalized, then we calculated mean values and different ratios, by comparing the biotinylation levels of samples labeled at different times (BAP-GFP_9h/BAP-GFP_3h = 3.34 ± 0.15 and BAP-Sox2_9h/BAP-Sox2_3h = 1.38 ± 0.12) or the biotinylation level differences between BAP-Sox2 and control BAP-GFP (BAP-Sox2/BAP-GFP = 389.66 ± 90.43 after 3 h of biotin pulse and BAP-Sox2/BAP-GFP = 147.07 ± 29.71 after 9 h of biotin pulse, Supplementary Table S5).

Thus, these data demonstrate that the PUB method allowed a quantitative estimate of in vivo interactions of the pluripotency transcription factors Sox2 and Oct4. This method allows detecting and quantifying PPI not only between proteins that interact through binding domains or colocalize in the nucleus but also between transcription factors assembling on closely spaced DNA binding sites, which results in the biotinylation of a BAP target linked to the protein of interest. This can provide further insights into the role of transcription factors in cells and their molecular mechanisms of action for developing therapeutic options.

### 3.5. Applications, Advantages, and Limitations of the Method

Comprehensive studies of protein localization to specific chromatin sites provide plenty of valuable information on the transcriptional control in cells and the relationships between transcription factors. Considering the methodological simplicity of the described method compared to other methods (e.g., split GFP [37]), this proximity-utilizing labeling method could be a useful tool to identify PPIs between known proteins. The PUB method, based on the use of the BAP/BirA system, has a number of advantages:

1. The design of BAP allows using both His-tag and streptavidin beads for the purification of the target proteins from non-specifically bound proteins, even under harsh conditions (high ionic strength of the solvent, presence of detergents, chaotropic agents).
2. A wide range of commercially available reagents can be used for the detection and purification of His-tagged and biotin-labeled target proteins.
3. Inside mammalian cells, the bacterial BirA enzyme does not biotinylate any endogenous protein, and conversely, the BAP is not recognized by the mammalian biotin ligase [32].
4. A vector is used that generates a BAP peptide with a wide temporal dynamic range of biotinylation linearity, which results in the isolation of a large number of even weakly interacting proteins. Alternative methods like BICON use Avitag, which gives a higher background biotinylation [38].
5. PUB [23] is oriented to the use of LC–MS/MS, which has higher sensitivity and accuracy than Western blot or other antibody-based detection methods. A similar method using biotin acceptor tags [32] cannot be combined with mass spectrometry.
6. The generation of a permanent covalent mark on one of the proteins of interest will allow one to bypass the limitations imposed by the extraction and purification procedures. Thus, the method should prove useful for the study of interactions that are otherwise difficult to detect by the Co-IP and tandem affinity purification (TAP) methods [30,39].

The PUB method is not without limitations and drawbacks:

1. The BirA enzyme is a 35kDa protein [40], significantly increases the size of the protein of interest, and could compromise its function and affect its PPI.
2. There is an apparent requirement to express at least low levels of an exogenous fusion protein [41].

## 4. Conclusions

Despite the fact that this method was developed as part of a joint Kazakh–French project during the internship of A.K. at the Gustave Roussy Institute (2007–2011), the work presented is the result of a transfer of technology and was completely carried out at the Kazakhstan National Center for Biotechnology. The method is simple to implement, and the basic equipment and most of the reagents used are standard for the average biochemistry, cell, and molecular biology laboratory. The presence of special equipment, such as a mass spectrometer, is also not critical, since the results can also be analyzed by Western blotting, which is especially important for laboratories with a small budget. The method is well reproducible, many experiments were performed in two (three) replicates, as well as using reagents, kits, and antibodies from various manufacturers. It should also be noted the flexibility of this method, which allows the analysis of the level of biotinylation of various cell fractions, such as nuclear and cytoplasmic fractions. In addition, there is the possibility of choosing various types of control in addition to GFP, including proteins localized in various cellular compartments. The results obtained in this work indicate that the method can be further used to analyze other transcription factors such as Nanog, Foxa, etc. A promising area of application of this method may also be the screening and selection of low-molecular-weight compounds that affect protein–DNA binding in a living cell. An interesting direction that we plan to take is the analysis and comparison of the post-translational modifications of Sox2 and Oct4 in the biotinylated fraction compared to those in the non-biotinylated fraction, the results of which can help understand the mechanisms of regulation of these proteins.

In conclusion, the experiments described suggest that a proximity-utilizing biotinylation technique based on BAP/BirA labeling of the target protein while proximal (interacting) can provide a useful alternative to conventional methods for analyzing PPIs. This technique provides an easy and accurate method of evaluation of protein–protein interactions (proximities) in vivo. In addition, we demonstrated that the PUB method can be applied in cases of DNA-dependent interactions involving pluripotency transcription factors, such as Sox2 and Oct4.The vectors which we designed for these experiments can be used to construct other expression plasmids by inserting the ORF of other genes and would thus serve as versatile reagents, not only for studies of protein–protein interactions but also for a wide variety of studies in proteomics and genomics using the advanced protein-labeling technology. After vector preparation, this protocol can be completed in seven working days.

## Supplementary Materials

The following are available online at https://www.mdpi.com/2218-273X/10/1/142/s1: Tables S1–S4: List of bound to Ni-sepharose proteins identified by LC–MS/MS, Table S5: Calculated ratios of biotinylation levels, protocols of transient transfection using FuGENE and calcium phosphate on six-well plates, Figure S1: Comparison of biotinylation levels in different fractions (nuclear vs. cytoplasmic).

## References

[1] Krallinger M., Leitner F., Rodriguez-Penagos C., Valencia A. (2008). Overview of the protein-protein interaction annotation extraction task of BioCreative II. Genome Biol..

[2] Keskin O., Tuncbag N., Gursoy A. (2016). Predicting Protein-Protein Interactions from the Molecular to the Proteome Level. Chem. Rev..

[3] Keskin O., Gursoy A., Ma B., Nussinov R. (2008). Principles of protein-protein interactions: What are the preferred ways for proteins to interact?. Chem. Rev..

[4] Ivanov A.A., Khuri F.R., Fu H. (2013). Targeting protein-protein interactions as an anticancer strategy. Trends Pharmacol. Sci..

[5] Wilson A.J. (2009). Inhibition of protein-protein interactions using designed molecules. Chem. Soc. Rev..

[6] Westermarck J., Ivaska J., Corthals G.L. (2013). Identification of protein interactions involved in cellular signaling. Mol. Cell. Proteom..

[7] Chen J., Sawyer N., Regan L. (2013). Protein-protein interactions: General trends in the relationship between binding affinity and interfacial buried surface area. Protein Sci..

[8] Rudolph J. (2007). Inhibiting transient protein-protein interactions: Lessons from the Cdc25 protein tyrosine phosphatases. Nat. Rev. Cancer.

[9] Karsenti E. (2008). Self-organization in cell biology: A brief history. Nat. Rev. Mol. Cell Biol..

[10] Boyer L.A., Lee T.I., Cole M.F., Johnstone S.E., Levine S.S., Zucker J.P., Guenther M.G., Kumar R.M., Murray H.L., Jenner R.G. (2005). Core transcriptional regulatory circuitry in human embryonic stem cells. Cell.

[11] Takahashi K., Tanabe K., Ohnuki M., Narita M., Ichisaka T., Tomoda K., Yamanaka S. (2007). Induction of pluripotent stem cells from adult human fibroblasts by defined factors. Cell.

[12] Takahashi K., Yamanaka S. (2006). Induction of pluripotent stem cells from mouse embryonic and adult fibroblast cultures by defined factors. Cell.

[13] Takahashi K., Yamanaka S. (2016). A decade of transcription factor-mediated reprogramming to pluripotency. Nat. Rev. Mol. Cell Biol..

[14] Yamanaka S. (2012). Induced pluripotent stem cells: Past, present, and future. Cell Stem Cell.

[15] Yamanaka S., Blau H.M. (2010). Nuclear reprogramming to a pluripotent state by three approaches. Nature.

[16] Chambers I., Tomlinson S.R. (2009). The transcriptional foundation of pluripotency. Development.

[17] Esch D., Vahokoski J., Groves M.R., Pogenberg V., Cojocaru V., Vom Bruch H., Han D., Drexler H.C., Arauzo-Bravo M.J., Ng C.K. (2013). A unique Oct4 interface is crucial for reprogramming to pluripotency. Nat. Cell Biol..

[18] Merino F., Ng C.K.L., Veerapandian V., Scholer H.R., Jauch R., Cojocaru V. (2014). Structural basis for the SOX-dependent genomic redistribution of OCT4 in stem cell differentiation. Structure.

[19] Tapia N., MacCarthy C., Esch D., Gabriele Marthaler A., Tiemann U., Arauzo-Bravo M.J., Jauch R., Cojocaru V., Scholer H.R. (2015). Dissecting the role of distinct OCT4-SOX2 heterodimer configurations in pluripotency. Sci. Rep..

[20] White M.D., Angiolini J.F., Alvarez Y.D., Kaur G., Zhao Z.W., Mocskos E., Bruno L., Bissiere S., Levi V., Plachta N. (2016). Long-Lived Binding of Sox2 to DNA Predicts Cell Fate in the Four-Cell Mouse Embryo. Cell.

[21] Goolam M., Scialdone A., Graham S.J.L., Macaulay I.C., Jedrusik A., Hupalowska A., Voet T., Marioni J.C., Zernicka-Goetz M. (2016). Heterogeneity in Oct4 and Sox2 Targets Biases Cell Fate in 4-Cell Mouse Embryos. Cell.

[22] Thomson M., Liu S.J., Zou L.N., Smith Z., Meissner A., Ramanathan S. (2011). Pluripotency factors in embryonic stem cells regulate differentiation into germ layers. Cell.

[23] Kulyyassov A., Shoaib M., Pichugin A., Kannouche P., Ramanculov E., Lipinski M., Ogryzko V. (2011). PUB-MS: A Mass Spectrometry-based Method to Monitor Protein-Protein Proximity in vivo. J. Proteome Res..

[24] Viens A., Mechold U., Lehrmann H., Harel-Bellan A., Ogryzko V. (2004). Use of protein biotinylation in vivo for chromatin immunoprecipitation. Anal. Biochem..

[25] Mechold U., Gilbert C., Ogryzko V. (2005). Codon optimization of the BirA enzyme gene leads to higher expression and an improved efficiency of biotinylation of target proteins in mammalian cells. J. Biotechnol..

[26] Betancourt L.H., Sanchez A., Pla I., Kuras M., Zhou Q.M., Andersson R., Marko-Varga G. (2018). Quantitative Assessment of Urea in-Solution Lys-C/Trypsin Digestions Reveals Superior Performance at Room Temperature over Traditional Proteolysis at 37 degrees C. J. Proteome Res..

[27] Villar-Garea A., Imhof A. (2006). The analysis of histone modifications. BBA-Proteins Proteom..

[28] Robin P., Fritsch L., Philipot O., Svinarchuk F., Ait-Si-Ali S. (2007). Post-translational modifications of histones H3 and H4 associated with the histone methyltransferases Suv39h1 and G9a. Genome Biol..

[29] Shoaib M., Kulyyassov A., Robin C., Winczura K., Tarlykov P., Despas E., Kannouche P., Ramanculov E., Lipinski M., Ogryzko V. (2013). PUB-NChIP-“in vivo biotinylation” approach to study chromatin in proximity to a protein of interest. Genome Res..

[30] Wang J., Rao S., Chu J., Shen X., Levasseur D.N., Theunissen T.W., Orkin S.H. (2006). A protein interaction network for pluripotency of embryonic stem cells. Nature.

[31] Slavoff S.A., Liu D.S., Cohen J.D., Ting A.Y. (2011). Imaging protein-protein interactions inside living cells via interaction-dependent fluorophore ligation. J. Am. Chem. Soc..

[32] Fernandez-Suarez M., Chen T.S., Ting A.Y. (2008). Protein-protein interaction detection in vitro and in cells by proximity biotinylation. J. Am. Chem. Soc..

[33] Chen I., Howarth M., Lin W., Ting A.Y. (2005). Site-specific labeling of cell surface proteins with biophysical probes using biotin ligase. Nat. Methods.

[34] Hymer W.C., Kuff E.L. (1964). Isolation of nuclei from mammalian tissues through the use of triton X-100. J. Histochem. Cytochem..

[35] Mir R.A., Bele A., Mirza S., Srivastava S., Olou A.A., Ammons S.A., Kim J.H., Gurumurthy C.B., Qiu F., Band H. (2016). A Novel Interaction of Ecdysoneless (ECD) Protein with R2TP Complex Component RUVBL1 Is Required for the Functional Role of ECD in Cell Cycle Progression. Mol. Cell. Biol..

[36] Puri T., Wendler P., Sigala B., Saibil H., Tsaneva I.R. (2007). Dodecameric structure and ATPase activity of the human TIP48/TIP49 complex. J. Mol. Biol..

[37] Cabantous S., Nguyen H.B., Pedelacq J.D., Koraichi F., Chaudhary A., Ganguly K., Lockard M.A., Favre G., Terwilliger T.C., Waldo G.S. (2013). A New Protein-Protein Interaction Sensor Based on Tripartite Split-GFP Association. Sci. Rep. UK.

[38] Lau P.N., Cheung P. (2013). Elucidating combinatorial histone modifications and crosstalks by coupling histone-modifying enzyme with biotin ligase activity. Nucleic Acids Res..

[39] Li Y.F. (2011). The tandem affinity purification technology: An overview. Biotechnol. Lett..

[40] Weaver L.H., Kwon K., Beckett D., Matthews B.W. (2001). Corepressor-induced organization and assembly of the biotin repressor: A model for allosteric activation of a transcriptional regulator. Proc. Natl. Acad. Sci. USA.

[41] Kulyyassov A., Ramanculov E. (2018). Application of Impact II high resolution quadrupole Time of-flight (QTOF) instrumentation in shotgun proteomics. Eurasian J. Appl. Biotechnol..

